# Histopathology of Cutaneous Leishmaniasis Caused by *Leishmania donovani* in Sri Lanka

**DOI:** 10.1155/2020/4926819

**Published:** 2020-05-02

**Authors:** Harshima Wijesinghe, Nayana Gunathilaka, Saveen Semege, Nishantha Pathirana, Nuwani Manamperi, Chandu de Silva, Deepika Fernando

**Affiliations:** ^1^Department of Pathology, Faculty of Medicine, University of Colombo, Sri Lanka; ^2^Department of Parasitology, Faculty of Medicine, University of Kelaniya, Sri Lanka; ^3^Army Preventive Medicine & Mental Health Services, Army Headquarters, Sri Jayawardenepura, Sri Lanka; ^4^Army Hospital, Colombo, Sri Lanka; ^5^Department of Parasitology, Faculty of Medicine, University of Colombo, Sri Lanka

## Abstract

Cutaneous leishmaniasis (CL) is a neglected tropical disease that is gaining importance in Sri Lanka and internationally. The clinical presentation, pathology, and method of parasite elimination in CL vary according to the species. *Leishmania donovani* is the causative organism for leishmaniasis in Sri Lanka. This collaborative cross-sectional study describes the clinicopathological features of cutaneous leishmaniasis among personnel of the tri-forces serving in the North and East of the country. The histology of fifty cases of CL confirmed by at least two methods (slit skin smear, lesion aspirate, tissue impression, and histology) was reviewed. The parasitic load was assessed semiquantitatively. The histological features were correlated with the clinical presentation and organism load. The majority (89.8%; *n* = 44) presented with a single lesion mostly located in the upper limb (69.4%). The lesion types included papule (34.7%), nodule (32.7%), and an ulcer (30.6%). The evolution time of lesions averaged 31.55 weeks. Epidermal changes were observed in 49 of the biopsies and included hyperkeratosis (90.0%; *n* = 45), acanthosis (44.0%; *n* = 22), atrophy (34.0%; *n* = 17), and interface change (66%; *n* = 33). Dermal changes were seen in all cases and were characterized by a lymphohistioplasmacytic inflammatory infiltrate of variable intensity with ill-formed granuloma in 19 cases (38%) and well-formed epithelioid granulomas in 22 cases (44%). Focal necrosis was present in 20% (*n* = 10). *Leishmania* amastigote forms were observed in 88% (*n* = 44). Transepidermal elimination (*P* = 0.025), granuloma (*P* = 0.027) formation, and type of lesion (*P* = 0.034) were significantly associated with the organism load. Granuloma formation was associated with a reduction in organism load, indicating that the macrophage activation played an important role in the control of the organism.

## 1. Background

Leishmaniasis, a neglected tropical disease, is caused by the intracellular protozoan of the genus *Leishmania*. Of the three main forms of clinical presentations seen with leishmaniasis, cutaneous leishmaniasis (CL) is the most common with an estimated 600,000 to one million new cases occurring worldwide annually [1]. The estimated global burden of this disease is believed to be higher than the reported numbers due to underreporting, under surveillance, and inadequate case detection techniques [2]. Since 1993, the geographical distribution of leishmaniasis is reported to have expanded significantly in the world, with a concomitant sharp increase in the number of cases [3] and emerging new disease foci [4]. Several countries have experienced epidemics including South Asian countries of Nepal, Bangladesh, and India [5, 6]. The first locally acquired case of CL was reported in Sri Lanka in 1992 [7], and it is currently an established parasitic disease in the country. *Leishmania donovani* MON-37 has been reported to be the causative organism of both cutaneous and visceral leishmaniasis in Sri Lanka [8, 9]. CL presents a spectrum of clinical and histopathological manifestations that encompass different morphological states such as nodule, plaque, and ulcer, and which may persist as a chronic lesion or heal with a scar, depending on the infecting parasite species [10]. The histopathological spectrum of changes seen in cutaneous leishmaniasis ranges from a diffuse infiltrate of macrophages, lymphocytes, and plasma cells to ill-defined granuloma to granulomatous inflammation with well-defined granuloma [11]. A single study conducted in Sri Lanka based on 31 skin biopsies described that lesions of CL could be classified into four histological groups I-IV that were similar to that of the spectrum seen in leprosy ranging from lepromatous to tuberculoid leprosy. The histological groups from I-IV showed a significant inverse relationship with the mean parasitic index [12]. Studies conducted elsewhere too have shown that organism load decreases progressively with the development of granulomatous inflammation [11] indicating that the development of granuloma is associated with increased control of the organism. Elimination of the parasite in cutaneous leishmaniasis has been thought to involve two mechanisms, activation of intact macrophages and a necrotizing process that destroys the macrophage with the parasite it contains [13–17]. Most studies have shown the latter to be the more efficient mechanism for parasite elimination [14, 18, 19]. However, a study conducted in Sri Lanka showed that necrosis was not a prominent feature and the main mode of parasite elimination was thought to be macrophage activation giving rise to epithelioid granulomata [12]. Currently, there are about 20 species of *Leishmania* that may cause CL. The clinical presentations, pathology, and method of parasite elimination vary according to the species. Therefore, understanding the immune mechanisms required for the control of infection within the varied tissue microenvironments that contain *Leishmania* is a key factor for developing effective vaccines and therapeutics. Evaluation of the histopathological features in a Sri Lankan population would facilitate a better understanding of host-parasite interaction. There is currently only one published study on the histopathology of CL due to *L. donovani* which is the causative organism of leishmaniasis in Sri Lanka [12]. Hence, the present study is aimed at describing the histopathological features of skin biopsies from patients with CL in Sri Lanka and correlating the histopathological characteristics with the organism load and clinical presentation.

## 2. Methods

### 2.1. Study Setting and Design

This was a collaborative cross-sectional descriptive study conducted by the Departments of Pathology and Parasitology of the Faculties of Medicine of the Universities of Colombo and Kelaniya and the Sri Lanka Army Medical Corps from November 2018 to November 2019. Clinical material collected from skin lesions of personnel of the tri-forces serving in the North and East of the country with features suspicious for CL was evaluated for the presence of *Leishmania* organisms by slit skin smear, lesion aspirate, tissue impression, and histology. All cases in which leishmania organisms were detected by at least two of the methods (slit skin smear, lesion aspirate, tissue impression, and histology) were included. Cases in which tissue was unsuitable for histological analysis due to severe crushing or autolysis were excluded. Ethical clearance for the study was obtained from the Ethical Review Committee of the Faculty of Medicine, University of Kelaniya, Ragama, Sri Lanka (P/94/02/2019). Permission to conduct the study was also obtained for the administration of the Sri Lanka Army.

### 2.2. Sample Collection

Two punch biopsies, each measuring 2–3 mm, were obtained from the lesion under local anaesthesia under aseptic conditions. An impression smear was obtained from the one biopsy which was thereafter fixed in 10% formalin for routine histopathological processing. Impression smears made on glass slides were air-dried, fixed in methanol, stained with Giemsa, and examined under a light microscope for the presence of parasites.

Slit skin smears were prepared by making a nick at the active edge of the lesion. Material from the gaped walls of the nick was scraped using the blunt side of the scalpel blade, and a thin skin smear was prepared. If the lesion had a scab, it was removed, and tissue material was scraped from the base of the lesion [4] to prepare the smear. Smears from fine-needle aspirates were obtained by injecting 0.1-0.2 ml sterile saline into the edge of the lesion with a 23 G needle fixed to a 2 ml syringe and applying suction while performing rotator to and fro movement. The slides prepared from skin scrapes and FNA were air-dried, fixed in methanol, followed by staining with Giemsa, and examined under oil emersion using a stereomicroscope for the presence of oval-shaped amastigotes with the characteristic dot and dash appearance. The sides were cross-checked by a consultant parasitologist as a quality control procedure.

### 2.3. Evaluation of Light Microscopy

All biopsies from the lesions clinically suspicious of CL were evaluated by two histopathologists at the Department of Pathology, Faculty of Medicine, Colombo. All tissue samples were dehydrated, cleared, embedded in paraffin, cut into 4–5 *μ*m thick sections, and stained with Haematoxylin and Eosin. Giemsa stain was performed in cases where the organisms were not detected on Haematoxylin and Eosin. The sections were assessed for histological features found in the epidermis and the dermis. The epidermis was evaluated for the presence of ulceration, crusting, transepidermal elimination, intraepidermal neutrophils, hyperkeratosis, parakeratosis, acanthosis, atrophy, spongiosis, exocytosis, and interface change. The dermis was evaluated for the intensity of inflammatory infiltrate, as well as the presence of different cell types in the infiltrate, the formation of granuloma, and the parasite load.

The inflammatory infiltrate in the dermis was categorized as mild, moderate, and extensive. A semiquantitative analysis of the constituting inflammatory cells (lymphocytes, macrophages, plasma cells, neutrophils, and eosinophils) was performed.

Based on the semiquantitative assessment, the infiltrate was categorized as macrophage predominant, lymphocyte predominant, predominantly lymphohistiocytic, and predominantly lymphoplasmacytic and having equal numbers of lymphocytes, plasma cells, and histiocytes.

The presence of necrosis was categorized as diffuse, focal or individual cell necrosis, and absent. Multinucleated giant cells, vasculitis, and neuritis were documented as being present or absent. The presence of granulomas was documented as absent, ill-defined, and well-defined. The organisms were quantified based on the Ridley parasitic index [15], and the overall histological pattern was categorized according to the modified Ridley's criteria [16]. The histological features were correlated with the nature of the lesion (patch, nodule, and plaque), the duration of the lesion, and organism load. The data analysis was conducted using the SPSS software package (version 23).

## 3. Results

Seventy-eight soldiers underwent further investigation for cutaneous lesions suspicious of leishmaniasis. The CL organisms were detected by at least one of the methods used in 63 cases (80.7%). Ten cases were positive only on histology, one only on slit skin smear, and one only on lesion aspirate. Therefore, these 12 cases were excluded. One case was excluded because the tissue was poorly preserved and not suitable for further histological evaluation. The remaining 50 cases were included in the study.

### 3.1. Clinical Features

Of the 50 patients with confirmed CL, only two were female (4%). Their age ranged from 19 to 54 years with an average age of 32.24 years. The evolution time of lesions varied from 3 to 660 weeks with an average of 31.55 weeks. The majority (89.8%; *n* = 44) presented with a single lesion, while four presented with two lesions and one with three lesions. The lesions were mostly located in the upper limb (69.4%), followed by back (10.2%), and lower limbs (8.2%). The lesion types included papule (34.7%), nodule (32.7%), and ulcer (30.6%). Only one patient presented with a plaque lesion.

### 3.2. Pathological Features: Epidermal Changes

The histopathological analysis from the 50 skin biopsies of patients with clinical and laboratory of localized CL showed morphological alterations in both, epidermis and dermis. Alterations of the epidermis were observed in 49 skin biopsies. Hyperkeratosis was present in 45 (90.0%) of the biopsies with it being the only epidermal change seen in five cases (10.0%). Acanthosis was present in 22 (44.0%) cases, while the epidermis was atrophic in 17 (34.0%). The majority (66%; *n* = 33) of biopsies showed interface change with varying degrees of basal cell degeneration and pigmentary incontinence. Other changes included parakeratosis in 42.0% (*n* = 21), spongiosis and exocytosis in 34.0% (*n* = 17), and ulceration in 14.0% (*n* = 7). Seven of the biopsies (14%) showed transepidermal elimination of amastigotes, while crusting and intraepidermal neutrophils were present in 12.0% (*n* = 6)) and 26% (*n* = 13), respectively (Table 1).

### 3.3. Pathological Features: Dermal Changes

**Figure 1:**
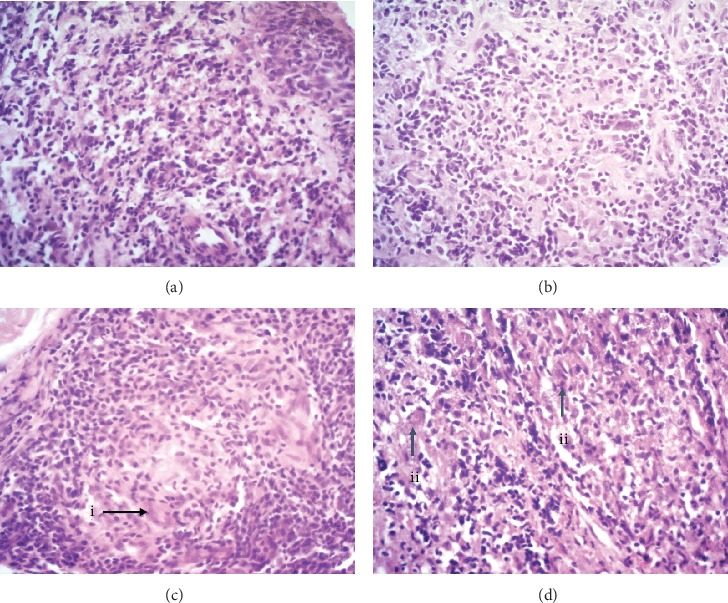
The spectrum of histopathological changes seen in the dermis (haematoxylin and eosin stain ×400): (a) diffuse lymphohistioplasmacytic infiltrate, (b) ill-defined granuloma, (c) well-defined granuloma with (i) multinucleated giant cells, and (d) diffuse lymphohistioplasmacytic infiltrate showing (ii) *Leishmania* amastigotes within macrophages.

Dermal changes were seen in all cases and were characterized mainly by a lymphohistiocytic inflammatory infiltrate of variable intensity (Figure 1(a)). Associated plasma cells were seen in 90% (*n* = 45). The inflammatory infiltrate was intense in 58% (*n* = 29), moderate in 32% (*n* = 42), and mild in 10% (*n* = 5) of the cases. It was characterized by a predominance of histiocytes in 44%, followed by lymphocytes in 14%, and comprised approximately equal numbers of lymphocytes, plasma cells, and histiocytes in 22% (*n* = 11). Neutrophils were seen in 34 cases (68%), while eosinophils were seen in fewer numbers and less frequently (14%; *n* = 7). Granuloma formation was present in 41 cases with ill-formed granuloma in 19 cases [38%] (Figure 1(b)) and well-formed epithelioid granulomas in 22 cases (44%) (Figure 1(c)). Multinucleated giant cells were seen in 26% (*n* = 13) and focal necrosis area in 20% (*n* = 10). None of the lesions showed vasculitis, but perineural inflammation was present in 10% (Table 2).

### 3.4. Detection of the Parasite

*Leishmania* amastigote forms were observed in 66% (*n* = 33) of HE-stained histological sections and seen only on Giemsa in 11 (22%) cases (Figure 1(d)). The parasite load ranged from 0 to 6 on the Ridley parasitic index (Table 3).

Most of the lesions were of Modified Ridley index (RMI) group 2 (38%; *n* = 19) or group 3 (34%; *n* = 17). One case in which histology was negative for organisms showed a perivascular chronic inflammatory infiltrate with no evidence of granuloma, so it did not fit into any of the RMI categories (Table 4). In patients with CL lesions of 24 weeks or less (early lesions), 85.7% were positive and 14.3% were negative; and in patients with lesions of more than 24 weeks of infection (late lesions), all seven lesions were positive.

### 3.5. Correlation of Clinicopathological Features and Organism Load

The clinical presentation (the type of lesion, duration of the lesion), epidermal changes, nature of dermal infiltrate, and presence of granuloma and RMI were correlated with the RPI using chi-squared analysis. Transepidermal elimination (*P* = 0.025), granuloma (*P* = 0.027) formation, and type of lesion (*P* = 0.034) were significantly associated with the organism load. Higher parasitic loads of RPI = 4 and above showed a positive association with transepidermal elimination and a negative association with well-defined granuloma. All lesions presenting as nodules showed organisms on histology, while the single lesion presenting as a plaque was negative for organisms. Of the biopsies that were negative for organisms on histology, apart from the single lesion that showed nonspecific perivascular inflammation, all other biopsies showed well-defined epithelioid granuloma. None of them showed exocytosis or spongiosis. All were from early lesions (<24 weeks). Lesions with a low RPI (RPI = 1 − 3) were more likely to be of RMI group 3 than RMI group 1, whereas lesions with a high RPI (RPI = 4 or above) were more likely to be of RMI group 1 than 3 (*P* < 0.001).

The 12 cases which were excluded, since organisms were detected only on one of the four detection methods, were reviewed. The two cases that had shown organisms only in slit skin smear and only in tissue impression had both been scored as plus 1. Of the 10 biopsies showing *Leishmania* parasite, 3 showed an RPI of 1 (1-10 organisms per standard) and 7 showed an RPI of 2 (11-100 organisms per standard section).

## 4. Discussion

Material from 78 patients with suspected CL was evaluated. The diagnosis was confirmed as CL, based on the positivity for amastigotes in at least two of the diagnostic methods used in 64.1% (50/78) of biopsies. When numerous amastigotes are present in a biopsy, cutaneous leishmaniasis is easy to diagnose. However, in the latter stages of the cutaneous infection when granulomas predominate and parasite-filled histiocytes gradually disappear, it becomes more difficult. Occasionally, the histopathological analysis fails to detect amastigotes even in early lesions [20]. In the current study too, the six biopsies in which organisms were not detected on histology were classified as early lesions of less than 24-week duration. The numbers of late lesions were small, and therefore, although all seven showed organisms on histological examination, this was not statistically significant.

In this study, the 12 cases were excluded since organisms were present in only one of the diagnostic methods used. All 12 cases showed a low parasitic load. CL is usually diagnosed on material obtained from patients with clinically suspicious lesions. Many methods including serology, slit skin smear, culture, histology, and polymerase chain reaction (PCR) are used in the diagnosis of cutaneous leishmaniasis [21]. Microscopic identification of the parasite has superior sensitivity to that of culture [21]. In difficult cases with occasional organisms, the availability of a reliable tool to identify the parasite would be extremely useful. While the inclusion of molecular techniques such as PCR has greatly improved the sensitivity of diagnosis, they are costly and not readily available. Immunohistochemistry is emerging as a new tool in the identification of the *Leishmania* organism, with studies showing that Cd1a immunostaining is useful in the detection of *Leishmania* organisms [22–25]. This has not been assessed in *L. donovani* species and may prove to be a useful adjunctive test in equivocal cases and cases of visceral leishmaniasis.

Morphological analysis of the 50 lesions with confirmed CL showed a variable parasite load (discrete to intense) and inflammatory infiltrates of variable intensity. Inflammatory infiltrates were characterized by a macrophage predominance, followed by lymphocytes and plasma cells. The epidermis showed both acanthosis and atrophy, accompanied by hyperkeratosis, parakeratosis, spongiosis, exocytosis, and interface changes. Other studies too have demonstrated epidermal changes such as exocytosis in CL [26, 27], highlighting the importance of the epidermis in the immunoregulation of the disease. In addition, lesions demonstrated the presence of ulcers, focal necrosis, and both ill-defined and well-organized granulomas. The presence of well-organized granulomas with multinucleated giant cells is a factor related to the immune system's attempt to eliminate parasites, and the organism load has also been shown to decrease progressively with the development of granulomatous inflammation [12], indicating that the development of granuloma is associated with increased control of the organism. In this study too, well-defined granuloma formation showed a positive association with a low organism load.

The histopathological and immunological host response to the infection caused by parasites of the genus *Leishmania* depends largely on the infecting parasite [28, 29]. In this study, three main morphological patterns were identified: a diffuse lymphohistioplasmacytic infiltrate, ill-defined histiocytic granuloma, and epithelioid granulomatous inflammation. This is similar to the pattern described by Azadeh et al. in 1985 [11]. The histological spectrum of CL has been classified variably in different studies. Venkataram et al. reported four histological patterns [20]. A more recent study conducted in Panama described three main histopathological patterns: a lymphohistiocytic inflammatory response, a lymphoplasmacytic response, and a granulomatous response [27].

Kurban et al. and Mansour et al. have suggested two histological patterns in biopsies of patients with CL: the first one corresponding to lesions of less than one-year duration associated with a diffuse infiltrate, and the second one corresponding to lesions of more than one-year duration associated with granulomatous infiltrate [30, 31]. In our study, however, we were not able to demonstrate a statistically significant relationship between the duration of the lesion and morphological pattern, with both early and late lesions demonstrating all three patterns of inflammation Well-defined granulomas were seen in 22 of the lesions, which included both early and late lesions. Well-defined granulomas were seen in a lesion of 660 weeks and a lesion of 4 weeks. Similarly, a diffuse infiltrate was seen in a late lesion of 78 weeks as well as lesions of 4 weeks. Although we were not able to demonstrate a correlation between the duration of the lesion and granuloma formation, well-defined granuloma showed a negative association with the parasite load, indicating that it was the immune response that has a greater correlation with the organism load than the duration of the lesion.

Activation of intact macrophages and a necrotizing process that destroys the macrophage with the parasite it contains are the two main methods of parasite elimination. In both the current study and the previous study conducted in Sri Lanka, necrosis was not a prominent feature and was seen focally in a minority of cases. However, the macrophage was the predominant cell in most cases. This is supportive of the theory that the main mode of parasite elimination in *L. donovani* infection is macrophage activation giving rise to epithelioid granulomata rather than necrosis [12]. Studies have shown T cell mediated IFN-*γ* and TNF-*α* activate parasite-infected macrophages for parasite destruction [32]. Studies in Sri Lanka showed that the mean percentage of T cells increased with the formation of granuloma and a reduction in parasite counts [12]. Additionally, a Sri Lankan study in which skin biopsies were analyzed for in situ cytokine expression of T helper cell cytokines showed a prominent T helper 1 response with increased IFN-*γ* levels in healing lesions [33], supporting the hypothesis that macrophage activation plays a key role in parasite elimination in Sri Lanka since IFN-*γ* is a potent activator of macrophages.

At present, there is no vaccine for the use against leishmaniasis and therapeutic options are limited due to factors such as drug toxicity, long treatment courses, challenging routes of drug administration, and instability of drugs in hot climates [34]. An understanding of the immune mechanisms required for the control of infection within the varied tissue microenvironments that contain Leishmania is a key factor for developing effective vaccines and therapeutics. Evaluation of the histopathological features indicates that macrophage activation appears to play a major role. Further characterization of the host immune response in a Sri Lankan population would facilitate a better understanding of host-parasite interaction and mechanisms of effective elimination of the parasite by the host.

## 5. Conclusions

In conclusion, the histopathological changes observed in localized CL caused by *L. donovani* in Sri Lanka were characterized by a variably intense inflammatory infiltrate with three main histological patterns: a diffuse lymphohistioplasmacytic infiltrate, ill-defined granuloma, and epithelioid granulomatous inflammation. The macrophage was the predominant cell in most cases. Necrosis was not a prominent feature. Organisms were present even in some late lesions of over 24-week duration, and the presence of granuloma did not show a relationship with the duration of the lesion. However, well-formed granuloma formation was associated with a reduction in organism load, indicating that the macrophage activation played an important role in the control of the organism.

## Figures and Tables

**Table 1:** Epidermal changes of lesions among patients with cutaneous leishmaniasis.

Epidermal change	Present	Absent
Ulceration	7 (14%)	43 (36%)
Transepidermal elimination of amastigotes	7 (14%)	43 (36%)
Hyperkeratosis	45 (90%)	5 (10%)
Parakeratosis	21(42%)	29 (58%)
Acanthosis	22(44%)	28 (56%)
Atrophy	17 (34%)	33 (66%)
Spongiosis	17 (34%)	33 (66%)
Exocytosis	17 (34%)	33 (66%)
Interface change	33 (66%)	17 (34%)
Crusting	6 (12%)	44 (88%)
Intraepidermal neutrophils	13 (26%)	37 (74%)

**Table 2:** Dermal changes of lesions among patients with cutaneous leishmaniasis.

Dermal change		Category	Number (%)
Type of inflammatory cells	Lymphocytes	Absent	0 (0)
		Fewer in number to other cells	3 (6)
		Equal in number to other cells	40 (80)
		Dominates other cells	40 (80)
	Plasma cells	Absent	5 (10)
		Fewer in number to other cells	27 (54)
		Equal in number to other cells	18 (36)
		Dominates other cells	0 (0)
	Macrophages	Absent	0 (0)
		Fewer in number to other cells	3 (6)
		Equal in number to other cells	25 (50)
		Dominates other cells	22 (44)
	Neutrophils	Absent	16 (32)
		Fewer in number to other cells	32 (64)
		Equal in number to other cells	2 (4)
		Dominates other cells	0 (0)
	Eosinophils	Absent	43 (86)
		Fewer in number to other cells	7 (14)
		Equal in number to other cells	0 (0)
		Dominates other cells	0 (0)
	Multinucleated giant cells	Present	13 (26)
		Absent	37 (74)

Nature of inflammatory infiltrate		Macrophage predominant	22 (44)
		Lymphocyte predominant	7 (14)
		Lymphoplasmacytic	1 (2)
		Lymphohistiocytic	9 (18)
		Lymphohistioplasmacytic	11 (22)

Degree of inflammation		Mild	5 (10)
		Moderate	16 (32)
		Extensive	29 (58)

Necrosis		Absent	40 (80)
		Focal	10 (20)
		Diffuse	0 (0)

Neuritis		Absent	45 (90)
		Present	5 (10)

Granuloma		Absent	9 (18)
		Ill-defined granuloma	19 (38)
		Well defined granuloma	22 (44)

Pattern of inflammation		Diffuse infiltrate	9 (18)
		Ill-defined histiocytic infiltrate	19 (38)
		Well defined granuloma	22 (44)

**Table 3:** Categorization of cases according to Ridley's parasitic index.

Score	Parasitic load	No of cases (%)
6	More than, 100,000 parasites per standard section	1 (2)
5	10,100–100,000 parasites per standard section	0 (0)
4	1001–10,000 parasites per standard section	9 (18)
3	101–1000 amastigotes per standard section	10 (20)
2	11–100 amastigotes per standard section	17 (34)
1	1–10 amastigotes per standard section	7 (14)
0	No amastigotes	6 (12)
	Total	50 (100)

**Table 4:** Categorization of cases according to modified Ridley index.

Group	Morphology	Organisms	No of cases (%)
Group 1	Parasitized macrophages with variable lymphocytes and plasma cells	+	8 (16)
Group 2	Parasitized macrophages with lymphocytes, plasma cells, and ill-formed histiocytic granuloma	+	19 (38)
Group 3	A mixture of macrophages (with or without parasites), lymphocytes, plasma cells, and epithelioid granuloma	+/-	17 (34)
Group 4	Epithelioid granulomatous response (with or without Langhans type multinucleated giant cells) with a few lymphocytes and plasma cells but no amastigotes	—	5 (10)
Nonspecific chronic inflammation with no amastigotes			1 (2)
Total			50 (100)

## Data Availability

Data can be made available on request from the corresponding author at harshima@path.cmb.ac.lk.
